# Creating an impact, not an impression: *ESC Heart Failure* in its seventh year

**DOI:** 10.1002/ehf2.13636

**Published:** 2021-10-07

**Authors:** Stephan von Haehling, Gabor Foldes, Zoltan Papp, Stefan D. Anker

**Affiliations:** ^1^ Department of Cardiology and Pneumology, Heart Center University of Göttingen Medical Center Göttingen Germany; ^2^ German Center for Cardiovascular Research (DZHK), partner site Göttingen Göttingen Germany; ^3^ National Heart and Lung Institute Imperial College London London UK; ^4^ Heart and Vascular Center Semmelweis University Budapest Hungary; ^5^ Division of Clinical Physiology, Department of Cardiology, Faculty of Medicine University of Debrecen Debrecen Hungary; ^6^ HAS‐UD Vascular Biology and Myocardial Pathophysiology Research Group Hungarian Academy of Sciences Budapest Hungary; ^7^ Department of Cardiology, Berlin Institute of Health Center for Regenerative Therapies, German Centre for Cardiovascular Research partner site Berlin Charité Universitätsmedizin Berlin Germany

We started *ESC Heart Failure* as the first open access heart failure journal in September 2014, and we would have never guessed that this journal would produce such a great interest in the heart failure community. Indeed, the first ever article to be published in *ESC Heart Failure*—by this group of authors—already noted that ‘research in acute and chronic heart failure is busier than ever worldwide and demands more publishing space’.^1^ Obviously, our starting conditions were a little easier than for many other new journals with a grown‐up sister journal published already for decades, the *European Journal of Heart Failure*, that would allow transfer to the new journal for submissions that could not be published, mostly for lack of space. Soon, the journal was indexed in PubMed, and in 2018, the first ever Impact Factor was released for *ESC Heart Failure*, reaching 3.407. Despite increasing submission numbers expected to grow to 1500 this year alone, we are extremely pleased to say that the impact factor has increased to 4.411 when it was published in June this year.

Looking into PubMed, a search of ‘ESC Heart Fail [JO]’ yields 1,424 results. Papers cover a very broad range of heart failure research from basic research over to randomized controlled trials, systematic reviews, review articles, case reports as well as rationale & design papers, and subanalyses of large‐scale clinical trials. With an increasing submission rate and a decreasing acceptance rate that still is about 50% of all submissions, we always aim to broaden clinical and basic research understanding of heart failure in its largest sense. Not surprisingly, the best cited paper in *ESC Heart Failure* published in 2020 by Felicitas Escher *et al*. was a paper on the detection of SARS‐CoV‐2 genomes, which was published while the pandemic was still raging. These authors used endomyocardial biopsies of 104 patients with suspected myocarditis or unexplained heart failure to detect SARS‐Cov‐2 genomes by real‐time reversed transcriptase polymerase chain reaction and found that five samples contained SARS‐CoV‐2. These findings were suggestive of myocardial injury based in SARS‐CoV‐2 accumulation in the myocardium, ultimately leading to ubiquitous troponin increase.^2^ Other papers having received high citation rates are listed in *Table* *1*. The table also highlights the broad spectrum of topics covered by the Journal.

**Table 1:** Top 10 of most cited manuscripts published in 2020 in *ESC Heart Failure*

Author	Article title	Publication year
Escher *et al*.^2^	Detection of viral SARS‐CoV‐2 genomes and histopathological changes in endomyocardial biopsies	2020
Wang *et al*.^3^	Cardiomyocyte‐derived exosomal microRNA‐92a mediates post‐ischemic myofibroblast activation both in vitro and ex vivo	2020
Vallabhajosyula *et al*.^4^	Pulmonary artery catheter use in acute myocardial infarction‐cardiogenic shock	2020
Martín‐Garcia *et al*.^5^	Effectiveness of sacubitril–valsartan in cancer patients with heart failure	2020
Patel *et al*.^6^	Diffuse right ventricular fibrosis in heart failure with preserved ejection fraction and pulmonary hypertension	2020
Zannad *et al*.^7^	Efficacy and safety of sodium zirconium cyclosilicate for hyperkalaemia: the randomized, placebo‐controlled HARMONIZE‐Global study	2020
Salvioni *et al*.^8^	Gender and age normalization and ventilation efficiency during exercise in heart failure with reduced ejection fraction	2020
Michel *et al*.^9^	Cardiac biomarkers for the detection of cardiotoxicity in childhood cancer—a meta‐analysis	2020
Schramm *et al*.^10^	Comparing short‐term outcome after implantation of the HeartWare® HVAD® and the Abbott® HeartMate 3®	2020
Karaye *et al*.^11^	Incidence, clinical characteristics, and risk factors of peripartum cardiomyopathy in Nigeria: results from the PEACE Registry	2020

Of course, the impact factor has been a matter of debate since its first release in 1975 after being originally suggested by Eugene Garfield. To understand how the impact factor is devised, it needs to be considered that only articles are taken into consideration that have been published 2 years before publication of the respective impact factor, for example, in June this year, the 2020 impact factor was released taking into account all publications in *ESC Heart Failure* published in 2018 and 2019 and the citations to these manuscripts also gained in 2018 and 2019. This point also highlights the fact that the 2 year impact factor may be useful for research with rapid turnover such as heart failure but not for other areas. Real ‘impact’ may sometimes be better reflected by the 5 year impact factor that is also published by Clarivate Analytics—the current publisher of the journal impact factor after Thomson Scientific and Healthcare sold this part of their portfolio in 2018.^12^

Anyhow, as the editors of *ESC Heart Failure*, we are proud to have an impact factor above 4 giving us a solid foundation among other heart failure journals for future publications (*Table* *2*), and we continue to welcome manuscripts from the field of heart failure in its broadest sense.

**Table 2:** Journals dedicated to publishing heart failure‐related materials with their respective impact factor

Journal name	2020 impact factor
*European Journal of Heart Failure*	15.534
*JACC ‐ Heart Failure*	12.035
*Circulation ‐ Heart Failure*	8.790
*Journal of Cardiac Failure*	5.712
*ESC Heart Failure*	4.411
*Heart Failure Reviews*	4.214
*Heart Failure Clinics*	3.179

Dedicated heart failure journals and their impact factors.
